# Developing evidence-based health education resources: a participatory and iterative approach applied to the one health concept

**DOI:** 10.3389/fpubh.2026.1912836

**Published:** 2026-09-23

**Authors:** Laure Hermet, Pauline Coraci, Amelie Descamps, Pia Touboul-Lundgren

**Affiliations:** 1 Department of Public Health, Nice University Hospital, Nice, France; 2 UR2CA-RESPECT, Université Côte d’Azur, Nice, France; 3 Nice Academy, Ministry of National Education, Nice, France

**Keywords:** co-construction, educational resources, evidence-based, health education, one health, schools

## Abstract

**Introduction:**

The development of health education resources frequently relies on a top-down approach centered on scientific or institutional expertise, which may not always reflect the needs of end users. In a context marked by the emergence of the One Health concept and its forthcoming integration into French school curricula, the development of appropriate educational resources represents a major challenge. This study aimed to describe a participatory, iterative and co-constructive approach to the development of evidence-based educational resources within the e-Bug program.

**Methods:**

Resource development followed a five-step process: needs assessment based on an evidence and context review, curriculum analysis and focus groups with teachers; resource design; prototype optimization through a second series of focus groups; scientific and institutional validation; and school-based piloting. Findings from the school-based evaluation are not reported in the present methodological study and will be presented separately. Qualitative data guided successive adaptations of the resources throughout the development process.

**Results:**

The identified needs led to the development of modular educational resources, adapted to different educational levels and readily usable in the classroom. Teacher feedback primarily concerned active learning approaches, content contextualization, activity modularity and the support provided to teachers, leading to further refinement of the resources. Scientific and institutional validation strengthened the accuracy of the content and supported its alignment with school curricula and public health priorities.

**Conclusion:**

This study highlights the value of a participatory approach involving teachers, scientific experts and institutional stakeholders in the development of health education resources. Through this One Health case study, it provides an example of an evidence-based methodology designed to enhance the relevance, acceptability and usability of educational resources intended for school settings.

## 1 Introduction

Health education is a key lever for disease prevention and the promotion of health-enhancing behaviors throughout life (1). Knowledge, attitudes, and practices related to infection prevention, vaccination, hygiene, and antimicrobial use are shaped from an early age. In this context, schools provide a unique setting to develop health literacy among children and adolescents and to foster a better understanding of contemporary public health challenges (2).

Increasing attention has been devoted to the development of health education resources in response to emerging health, environmental, and societal challenges (3–5). Numerous initiatives now aim to support teachers in addressing complex topics such as antimicrobial resistance, climate change, zoonotic diseases, environmental health, and the prevention of infectious diseases. However, although frameworks for intervention development increasingly emphasize stakeholder engagement, iterative refinement, and implementation considerations, the methodological processes used to co-design, adapt, and validate educational resources are often insufficiently described in the published literature (6–9).

Educational resources are often developed using a top-down approach, relying primarily on scientific or institutional expertise. While such an approach is essential to ensure the accuracy of content, it may result in tools that are insufficiently adapted to real-world contexts, pedagogical constraints, or the expectations of end users. Several studies have highlighted the importance of involving relevant stakeholders early in the development process to improve the relevance, acceptability, feasibility, and uptake of educational interventions (8).

In this context, participatory and iterative approaches appear particularly relevant. By continuously involving key stakeholders: including teachers, scientific experts, educational institutions, and public health organizations, these approaches make it possible to integrate evidence-based knowledge, field-based needs, curriculum requirements, and public health priorities simultaneously. Despite growing interest in such approaches, few studies have described in detail how they are involved in the development of educational resources for schools, particularly when addressing emerging and multidisciplinary topics.

The One Health concept provides a particularly relevant example of this challenge (3, 10). Based on the recognition of the interconnections between human, animal, and ecosystem health, it draws upon knowledge from multiple disciplines and requires the integration of health, environmental, and societal issues. Now widely recognized as a key framework for addressing challenges such as antimicrobial resistance, zoonotic diseases, emerging infectious diseases, and the health impacts of climate change, One Health also represents a significant educational challenge. Its systemic and cross-cutting nature makes the development of accessible and age-appropriate educational resources for school settings particularly complex (11).

In France, interest in the One Health approach has increased considerably in recent years (12, 13). This momentum is reflected in its progressive integration into public policies addressing infection prevention and antimicrobial resistance, as well as its explicit inclusion in the future national school curriculum that will come into effect from September 2027 (14). In this context, the development of appropriate educational resources is essential to support the progressive appropriation of these concepts by both teachers and students.

The international e-Bug program, established in 2006 and coordinated in France by the Nice University Hospital, develops evidence-based educational resources on infections, their transmission, prevention, and treatment (15–17). Built on close collaboration between health professionals, educators, and public institutions, e-Bug provides a particularly relevant framework for documenting the processes involved in the development, adaptation, and validation of new educational resources addressing emerging public health priorities.

The aim of this article is to describe an evidence-based participatory and iterative approach to the development of health education resources, applied to the One Health concept within the e-Bug program. Through this case study, we analyze how the successive integration of scientific evidence, teachers’ needs, pedagogical constraints, institutional expectations, and public health priorities contributed to the development of resources adapted to the school context and intended to facilitate their future implementation.

## 2 Materials and methods

### 2.1 Project context and framework

The development of the One Health educational resources was conducted within the framework of the e-Bug program, an international health education initiative established in 2006 and coordinated in France by Nice University Hospital. The program aims to develop evidence-based educational resources to improve children’s and adolescents’ knowledge of infections, their transmission, prevention, and treatment, while also supporting teachers in updating their knowledge and integrating these topics into school curricula (18).

In response to the growing challenges posed by antimicrobial resistance, zoonotic diseases, environmental health, and climate change, e-Bug France initiated the development of new educational resources focused on the One Health concept. The development process followed a participatory and iterative approach involving teachers, scientific experts, institutional partners, and educational stakeholders.

Resource development was based on a co-construction approach centered on end users, involving teachers, scientific experts, and institutional partners throughout the design process in an iterative manner. Each stage aimed to integrate available evidence, educational constraints within school settings, user feedback, and emerging public health priorities in order to optimize the relevance, feasibility, acceptability, and usability of the resources produced.

The overall process took place between January 2025 and September 2026, with final adjustments made following the school-based piloting phase and national dissemination of the finalized resources scheduled for the beginning of the 2026-2027 school year in September 2026. The process comprised five successive stages: (1) needs assessment, (2) resource development, (3) prototype optimization, (4) scientific and institutional validation, and (5) school-based piloting (Figure 1).

**Figure 1:**
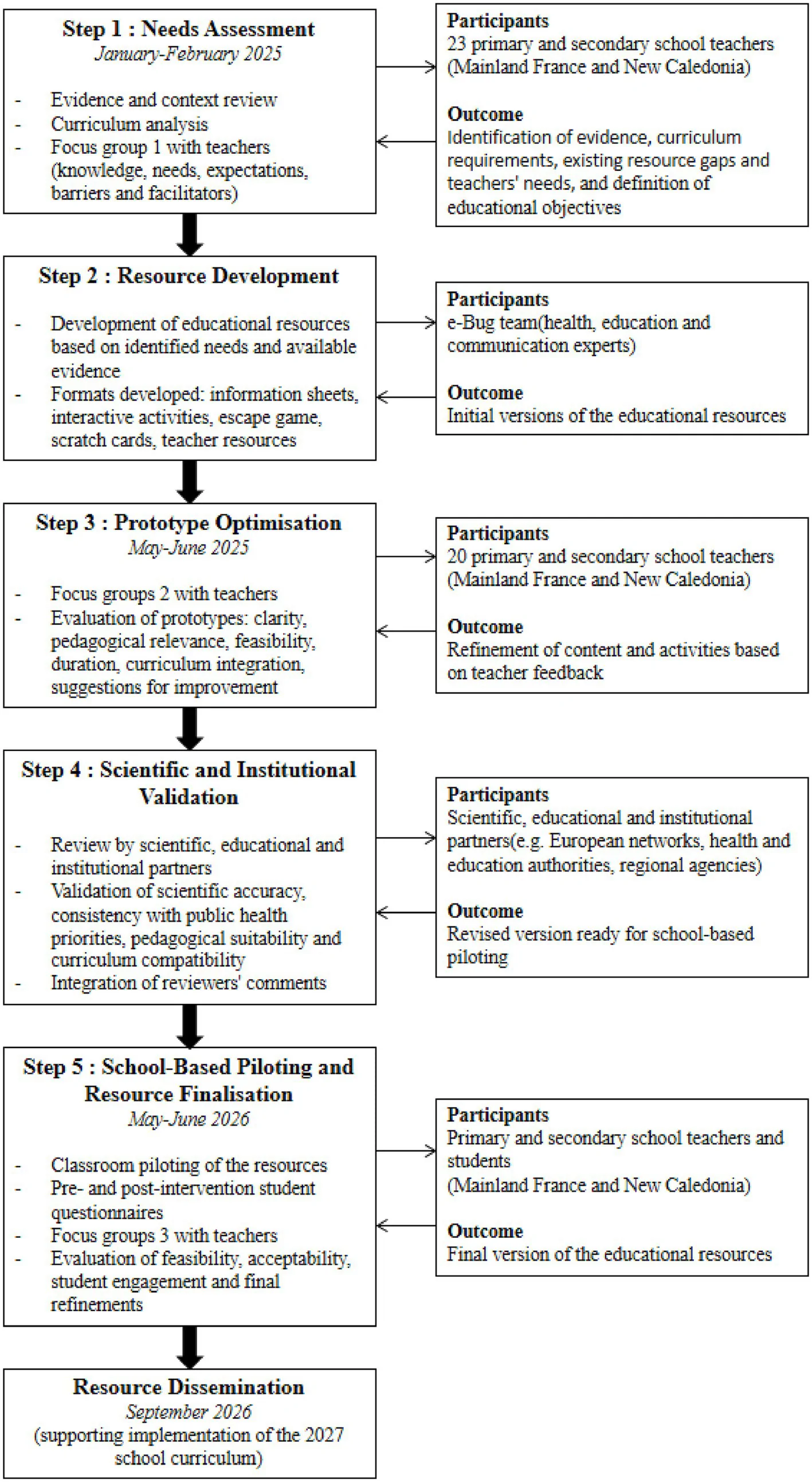
Participatory and iterative co-construction framework for the development of one health educational resources.

### 2.2 Step 1: needs assessment

#### 2.2.1 Evidence and context review and curriculum analysis

An initial evidence and context review was conducted to inform the development of the One Health educational resources. This exploratory review was not designed as a systematic literature review, but aimed to identify relevant and up-to-date scientific, epidemiological, educational, and contextual information. Scientific literature and French epidemiological and surveillance data, including data from Santé publique France, were reviewed to identify current public health priorities and ensure that the scientific content reflected the French health context. Where relevant, national and local epidemiological data and concrete examples were incorporated into the educational activities to make the resources more contextualized and meaningful for students.

In parallel, the French school curricula in force at the time of the study, as published in the official national curriculum documents (Bulletin officiel de l’Éducation nationale), were examined to identify opportunities for integrating One Health-related concepts across different educational levels and the learning objectives and competencies that the educational resources could support. Ongoing curriculum developments were also considered through exchanges with the French Ministry of National Education, including the forthcoming curricula strengthening the integration of the One Health approach.

Existing educational resources were explored through the French Ministry of National Education’s Eduscol platform and an exploratory web search for One Health educational resources. This was complemented by resources identified or reported by teachers during the exploratory focus groups. The purpose was to identify existing materials and potential gaps in educational resources providing an integrated One Health approach suitable for the French school context.

Information was selected according to its relevance to the One Health topics addressed, its scientific currency, its applicability to the French public health and school contexts, and its alignment with curriculum learning objectives. Findings from this evidence and context review were considered alongside the needs and constraints identified by teachers during the exploratory focus groups to define the educational objectives and guide the initial development of the resources.

A summary of the sources reviewed, their purpose, and their contribution to resource development is provided in Supplementary file 1.

#### 2.2.2 Exploratory focus groups with teachers

An initial exploratory phase was conducted between January and February 2025 involving 25 primary and secondary school teachers from mainland France and New Caledonia. Four focus groups were carried out to identify knowledge, needs, expectations, barriers, and facilitators related to teaching the One Health concept (11).

Discussions explored teachers’ baseline knowledge of infectious diseases, environmental issues, and the One Health approach, as well as their expectations and needs regarding potential educational resources, their perceptions of the place of these topics within school curricula, and the constraints likely to influence their implementation in the classroom.

Detailed methods and findings from the exploratory focus groups have already been published and served as the foundation for defining the educational objectives and guiding the development of the resources (11).

### 2.3 Step 2: resource development

The resources were developed based on the findings of the evidence and context review and curriculum analysis, together with the needs and constraints identified during the exploratory focus groups. These complementary sources were translated into educational objectives and functional specifications that guided resource development.

Particular attention was paid to adapting the content to different educational levels, promoting feasibility in real classroom settings, and maintaining alignment with French school curricula. Several complementary educational formats were developed to address the diversity of needs identified during the focus groups, including information sheets, interactive learning activities, knowledge-consolidation resources, and tools designed to promote student engagement.

Throughout the development process, some activities were also presented during awareness-raising events and training sessions involving school-aged audiences, members of the general public, and teachers. These preliminary uses provided informal feedback regarding the clarity of instructions, the level of engagement generated by the activities, and their feasibility, thereby contributing to successive refinements of the resources prior to formal evaluation.

### 2.4 Step 3: prototype optimization

Between May and June 2025, a second series of focus groups was conducted involving 20 primary and secondary school teachers from mainland France and New Caledonia to evaluate the initial versions of the resources. Participants were drawn from the same group of teachers who had participated in the initial exploratory focus groups (FG1) and were recontacted to review the resources developed on the basis of their previously identified needs. Teachers who were unable to attend the second focus groups were given the opportunity to provide individual feedback on the resources by email. Participants were invited to review the scientific content, educational objectives, proposed materials, and classroom implementation procedures. Discussions focused on content clarity, pedagogical relevance, feasibility, estimated duration, and compatibility with school curricula.

This second series of focus groups was conducted following the same methodological principles as the first series. Interviews were audio-recorded, transcribed verbatim. The transcripts were coded by one researcher using NVivo software and analyzed thematically (19). Given the formative purpose of this second series of focus groups, the analysis was primarily structured around the domains explored in the interview guide, including content, pedagogical relevance, curriculum alignment, classroom implementation, and barriers and facilitators to use the resources. Additional codes were developed inductively when issues emerged from the discussions that were not captured by these predefined domains. The coding and resulting categories were reviewed by the researcher who had observed the focus groups, and interpretations were discussed between the two researchers until consensus was reached. Suggested modifications were subsequently incorporated into a revised version of the resources. The focus group guide used for this prototype optimization phase is provided in Supplementary file 2.

### 2.5 Step 4: scientific and institutional validation

The revised resources were then submitted to a multidisciplinary review process involving scientific, institutional, and educational stakeholders. This phase included representatives from the Ministry of Health (French Directorate General for Health DGS), Santé publique France, Regional Centers for Antibiotic Stewardship (CRAtb), Regional Support Centers for the Prevention of Healthcare-Associated Infections (CPias), the Ministry of National Education (General Inspectorate for Education, Sport and Research IGÉSR), educational authorities from mainland France and New Caledonia, the French Ministry of Agriculture, the Provence-Alpes-Côte d’Azur Regional Health Agency (ARS), and several regional health promotion organizations.

The purpose of this stage was to assess the scientific accuracy and currency of the content, its consistency with national public health priorities and recommendations, its pedagogical suitability, and its compatibility with existing institutional and curricular frameworks. The revised resources were circulated to partner organizations by email and were also presented and discussed during an online meeting. Partners reviewed the resources according to their respective areas of expertise. Scientific and public health partners primarily reviewed the accuracy and currency of scientific and epidemiological content and its consistency with public health recommendations and priorities, while educational and institutional partners reviewed alignment with school curricula, learning objectives, pedagogical expectations, and relevant institutional frameworks. No standardized assessment grid was used, as the multidisciplinary process was intended to draw on the complementary expertise and responsibilities of the different partners involved.

Feedback received during this phase was collected by email and during the online meeting and subsequently reviewed by the e-Bug France project team, comprising a public health physician, a public health nurse, and a secondary-school life and earth sciences teacher. Recommendations were discussed within the project team and incorporated into successive versions of the resources. No conflicting recommendations requiring arbitration were identified during this process.

### 2.6 Step 5: school-based piloting and resource finalization

The revised resources were piloted in volunteer schools between May and June 2026.

This phase relied on a mixed-methods approach combining:

- Pre- and post-intervention knowledge assessment questionnaires administered to students, with the post-intervention questionnaire also including open-ended questions exploring students’ perceptions of the resources, including their overall experience, interest in the topic, aspects they appreciated or disliked, and suggestions for improvement.
- Evaluation of feasibility, acceptability, and implementation conditions by teachers.
- A third series of focus groups conducted following classroom implementation.

This evaluation aimed to document the feasibility and acceptability of the resources under real-world conditions, assess student engagement and knowledge acquisition, and identify any adaptations made during implementation as well as opportunities for further improvement. All data collected during this phase, including students’ perspectives, contributed to the development of the final version of the One Health educational resources. The detailed quantitative and qualitative findings from this school-based evaluation are outside the scope of the present methodological article and will be reported separately.

Data obtained from focus groups, scientific reviews, and institutional consultations were analyzed iteratively. Feedback covered complementary scientific, pedagogical, organizational, and institutional dimensions and was collectively reviewed by the e-Bug France project team before being translated into successive modifications of the resources. Modifications made to the resources were documented throughout the process to ensure traceability of successive adaptations.

### 2.7 Data sources and iterative development process

Resource development was informed by multiple complementary sources, including exploratory and evaluative focus groups with teachers, scientific reviews, institutional consultations, and feedback collected during awareness-raising activities and training sessions. Findings from each phase were reviewed by the project team and translated into successive modifications of the resources. This iterative process enabled the integration of scientific evidence, user feedback, pedagogical constraints, and institutional recommendations throughout resource development.

### 2.8 Ethical considerations

Participants received detailed information regarding the study objectives and procedures and provided written informed consent prior to participation. Data were anonymized during transcription and analysis. The study was conducted in accordance with the French Reference Methodology MR-004 governing research involving personal data and was registered on the Health Data Hub platform under number 23258975, March 25 2025.

## 3 Results

### 3.1 From identified needs to resource design principles

The initial evidence and context review identified several complementary elements relevant to the development of the One Health educational resources. The analysis of French school curricula identified multiple opportunities for addressing One Health-related topics across educational levels, as well as learning objectives and competencies that could be supported by the proposed activities. Ongoing curriculum developments further reinforced the relevance of explicitly integrating the One Health approach into educational resources.

The review of scientific, epidemiological, and surveillance data identified current public health issues and context-specific information that could be incorporated into the resources, including up-to-date French epidemiological data and, where relevant, concrete national or local examples. The exploration of existing educational resources did not identify materials providing an integrated One Health approach specifically adapted to the French school context. This finding was consistent with the needs subsequently expressed by teachers during the exploratory focus groups. A summary of the sources reviewed and their contribution to resource development is provided in Supplementary file 1.

The exploratory phase conducted with teachers identified several conditions considered essential for facilitating the integration of the One Health concept into school settings. Participants highlighted the need for resources that were readily usable in the classroom, adapted to different educational levels, aligned with curricula, scientifically reliable, based on up-to-date evidence, and supported by active learning approaches. They also expressed the need for support in appropriating a concept that was still relatively unfamiliar, while avoiding approaches likely to generate anxiety among students. These findings were published previously and served as the foundation for the development of the educational resources.

The main needs identified during this phase were translated into design principles that guided the entire development process (Table 1).

**Table 1:** Translation of needs identified during the exploratory phase into educational resource design principles.

Needs identified during FG1	Design principles adopted
Ready-to-use resources	Development of resources directly usable in the classroom
Active and engaging activities	Use of interactive and game-based learning approaches
Progressive learning pathway	Adaptation of content according to educational level
Visual supports	Integration of illustrations, diagrams, and graphical materials
Curriculum alignment	Explicit correspondence with curricular competencies and content
Need for scientific reassurance	Development of a teacher guide and scientific validation of content
Time constraints and diversity of school contexts	Development of flexible and adaptable resources

### 3.2 Initial development of educational resources

Based on these principles, several complementary resources were developed to address different dimensions of the One Health concept and respond to the diversity of pedagogical contexts encountered in schools.

The educational resources included information sheets on infectious diseases illustrating interactions between human, animal, and environmental health; an activity focusing on biocide use and its potential impacts on the environment and antimicrobial resistance; an escape game incorporating collaborative problem-solving approaches; an activity inviting students to design their own pandemic scenarios; and scratch cards designed to reinforce the acquisition of preventive behaviors (20). The resources were developed according to a modular approach, enabling teachers to use them either independently or in combination. Individual activities could therefore be extracted from the complete learning pathway and implemented autonomously depending on educational objectives, school level, or available teaching time. For example, activities embedded within the escape game could be used separately without requiring implementation of the full scenario. Preliminary testing conducted during awareness-raising activities and teacher training sessions confirmed the engagement potential of the proposed activities. Teachers participating in training sessions were invited to complete the activities themselves under conditions similar to those intended for students, allowing several practical adjustments to be identified prior to formal classroom piloting.

The resources were designed according to a complementary framework, enabling teachers to select the tools most appropriate to their teaching context while maintaining overall coherence around the One Health concept.

### 3.3 Resource optimization through teacher feedback

The second phase of focus groups enabled evaluation of the initial versions of the resources and identification of several areas for improvement. The analysis identified recurring categories of feedback relating to pedagogical approaches and student engagement, content accessibility and adaptation to different educational levels, curriculum alignment, and practical implementation in the classroom.

Teachers confirmed their interest in educational approaches promoting active student participation, the use of authentic data, and learning anchored in real-world situations. Resources were considered particularly relevant when they allowed students to construct knowledge themselves through observation, inquiry, and problem-solving activities.

Discussions also highlighted the need to simplify certain scientific concepts, improve the readability of some materials, and strengthen the alignment between the proposed activities and existing school curricula. Participants emphasized the importance of limiting the amount of information presented simultaneously to the students, providing visual resources, and offering different levels of complexity to facilitate adaptation to diverse student populations. Practical considerations included the need to accommodate different teaching time constraints and contexts, as well as the need for additional guidance to support teachers in addressing a relatively unfamiliar concept.

These findings resulted in several modifications to the resources prior to classroom piloting (Table 2).

**Table 2:** Main adaptations made to the educational resources following formative evaluation of the prototypes by teachers.

Teacher feedback	Adaptations implemented
Promote student knowledge construction rather than a top-down transmission of information	Strengthening of inquiry-based learning, information-seeking, and problem-solving activities
Certain One Health concepts appeared abstract or complex for students	Addition of concrete examples, case studies, and diagrams illustrating interactions between human, animal, and environmental health
Resources needed to accommodate varying time constraints and teaching contexts	Development of a modular structure enabling independent or combined use of activities
Importance of visual supports to facilitate understanding and engagement	Reinforcement of illustrations, diagrams, infographics, and graphical materials
Need to adapt content to different educational levels	Progressive differentiation of activities and content according to educational level
Difficulty identifying the place of One Health within existing curricula	Addition of explicit links to curriculum content and targeted competencies
Need for support when addressing a relatively unfamiliar concept	Development and enrichment of the teacher guide with scientific information, pedagogical guidance, and classroom implementation suggestions
Importance of activities promoting interaction and engagement	Integration of game-based and collaborative approaches (escape game, scratch cards, group activities)

### 3.4 Contribution of scientific and institutional validation

The revised resources were subsequently submitted to a validation phase involving scientific, educational, and institutional partners. This stage contributed to strengthening the scientific accuracy of the content, its consistency with public health messages, and its compatibility with school curricula. The partners consulted also contributed to strengthening the relevance of the resources in relation to contemporary One Health challenges, particularly in the fields of antimicrobial resistance, emerging infectious diseases, and environmental health.

The feedback received resulted in several additional refinements, including improvements to the scientific precision of specific content, updates to epidemiological data, clarification of key concepts, reinforcement of links with public health and educational policies, and the development of additional implementation supports for teachers. Institutional reviewers contributed not only to the scientific accuracy of the resources but also to their practical implementation by suggesting improvements related to teacher support materials, alignment with public health recommendations, accessibility of the resources, and links with existing public health tools.

### 3.5 Toward a participatory and iterative model for developing health education resources

A retrospective analysis of the process highlights a development model based on the successive integration of four complementary forms of expertise: teacher expertise, scientific expertise, institutional expertise, and expertise derived from real-world implementation. The combination of these different sources of information was intended to strengthen the scientific robustness of the content, its pedagogical relevance and potential feasibility in school settings, its acceptability among teachers, and its consistency with institutional expectations.

Furthermore, in accordance with the methodological approach adopted by the e-Bug program, resource development does not represent the final stage of the process. The resources subsequently underwent a school-based piloting phase involving quantitative evaluation of knowledge acquisition and students’ perceptions of the resources, qualitative evaluation with teachers, and ongoing collection of comments and suggestions for improvement during implementation and following online dissemination. This phase aims to document the feasibility, acceptability, and educational impact of the resources under real-world conditions. The quantitative and qualitative findings from this school-based evaluation are not reported in the present methodological article and will be reported in a separate publication.

The process developed within the One Health project therefore appears to represent a transferable model for the development of future health education resources intended for school settings.

## 4 Discussion

This study describes a participatory and iterative approach to the development of evidence-based health education resources, applied to the One Health concept within the e-Bug program. The successive integration of teachers’ needs, available scientific evidence, feedback obtained through formative evaluation of the resources, and institutional recommendations enabled the development of educational tools adapted to the school context. Our findings highlight the value of a co-construction approach involving future users throughout the development process in order to promote the relevance, feasibility, and acceptability of the resources produced.

Health education resources are still frequently developed using a top-down approach that relies primarily on scientific or institutional expertise. Although such an approach generally ensures the accuracy of content, it does not always account for the realities of educational settings, organizational constraints within schools, or the specific needs of teachers. In our study, the early involvement of teachers enabled the identification of several key factors influencing future resource uptake, including the need for classroom-ready materials, alignment with school curricula, flexibility across teaching contexts, and the use of active learning approaches. The successive consultation phases also allowed the resources to be progressively refined in response to time constraints, pedagogical expectations, and students’ levels of knowledge. In particular, teachers consistently emphasized the importance of active, interactive and student-centered learning approaches. This preference is consistent with previous evidence showing that active learning strategies can improve student engagement and learning outcomes compared with more traditional transmissive approaches (21, 22). These findings are consistent with previous studies highlighting the importance of stakeholder engagement in the development of educational interventions in order to improve their acceptability, relevance, and implementation conditions (8).

One of the main lessons emerging from this study is the complementarity of the different forms of expertise mobilized throughout the process. Teachers contributed their knowledge of educational realities and classroom constraints; the e-Bug team contributed to the scientific consistency of the content; and institutional partners helped guarantee alignment with public health priorities and existing curricular frameworks. This combination of expertise appears to be a central determinant of the overall quality of the resources produced. Beyond scientific accuracy, educational resources must also be usable, acceptable, and compatible with teachers’ professional practices. Our findings therefore suggest that the quality of a health education resource depends not only on the validity of its content but also on its ability to respond to the needs and constraints of its intended users.

The One Health concept provides a particularly relevant example for illustrating the value of this approach. Although it is now widely recognized as a key framework for addressing many contemporary public health challenges, its multidisciplinary and systemic nature makes its pedagogical translation inherently complex (10, 23). Findings from the exploratory phase revealed limited familiarity with the concept among teachers, together with a substantial need for pedagogical support (11). Within this context, co-construction supported the translation of a complex scientific concept into educational resources designed for school settings while preserving its conceptual richness. This issue is particularly relevant in the current French context, marked by the explicit integration of the One Health approach into future school curricula that will come into force from the 2027 academic year.

This study presents several strengths. First, it is based on a structured process involving successive cycles of needs assessment, development, optimization, and validation. Second, it mobilized a diverse range of stakeholders from the health, education, and public sectors, allowing complementary perspectives that are rarely combined in the development of educational resources. Finally, the e-Bug approach does not stop at resource development but systematically incorporates a subsequent school-based evaluation phase combining quantitative and qualitative methods. This articulation between development and evaluation represents one of the methodological characteristics of the program and contributes to the continuous improvement of the resources produced.

Several limitations should nevertheless be acknowledged. Teachers participating in the different phases were recruited on a voluntary basis and were likely more interested in health-related topics than the broader teaching population. Students were not directly involved in the initial co-construction stages, which primarily focused on curriculum alignment, learning objectives, pedagogical constraints, and classroom implementation and therefore relied mainly on teachers’ expertise. As students are the ultimate recipients of the resources, their absence from these early stages represents a limitation, particularly regarding the consideration of their perspectives on engagement and accessibility. However, students were directly involved during the school-based piloting phase, when they had the opportunity to experience the resources under classroom conditions. In addition to knowledge assessment, post-intervention questionnaires included open-ended questions on their perceptions of the resources and suggestions for improvement, which contributed to the final adjustments. The initial evidence and context review was exploratory rather than systematic and may therefore not have identified all relevant scientific evidence or existing educational resources. In addition, this study specifically documents the development of resources within the French educational context. The resources produced through the e-Bug program are intended to be made available to all partner countries within the network. However, their implementation in other contexts requires an adaptation process that takes into account the curricular, cultural, linguistic, and institutional specificities of each country. Finally, this study focuses on the resource development process rather than on educational outcomes, the evaluation of which will be addressed through subsequent analyses.

This study illustrates how the e-Bug development methodology can be mobilized to address emerging public health priorities. This is consistent with recent international recommendations highlighting education as a key component of strategies to address antimicrobial resistance and promote One Health literacy among future generations (24). The application of this approach to the One Health concept illustrates its potential to integrate new scientific knowledge, adapt it to the realities of educational settings, and translate it into educational resources that are compatible with both teachers’ expectations and institutional frameworks. In a context marked by the forthcoming integration of the One Health approach into French school curricula, this experience provides a concrete example of evidence-based educational resource development co-constructed with future users. More broadly, it highlights the value of involving stakeholders early and continuously in the development of educational resources intended for school settings.

## 5 Conclusion

The development of health education resources relies not only on the production of scientifically accurate content but also on their ability to address teachers’ needs, fit within the constraints of school settings, and align with public health priorities. Through the example of the One Health concept, this study describes a participatory and iterative approach involving teachers, scientific experts, and institutional partners throughout the development process. This approach supported the translation of a complex scientific concept into a set of evidence-based educational resources adapted to school settings and designed to be readily usable by their intended users. Beyond the specific case presented here, this work highlights the value of a structured co-construction methodology for the development of health education resources.

In a context characterized by emerging public health challenges and the increasing integration of health-related topics into school curricula, the early and continuous involvement of stakeholders appears to be a key lever for enhancing the relevance, acceptability, and future implementation of educational resources. The methodology presented in this study may provide a useful framework for the development of future evidence-based educational interventions addressing complex and multidisciplinary public health issues.

## Data Availability

The datasets presented in this article are not readily available because the datasets are not publicly available due to the qualitative nature of the data and the risk of participant identification. Access may be granted upon reasonable request to the corresponding author, subject to ethical approval. Requests to access the datasets should be directed to Laure Hermet, hermet.l@chu-nice.fr.
