# Supplementary material for: Developing evidence-based health education resources: a participatory and iterative approach applied to the one health concept
Source: Front Public Health. 2026 Sep 23;14:1912836. doi: 10.3389/fpubh.2026.1912836 (PMC13642583; doi:10.3389/fpubh.2026.1912836)
Supplement: Supplementary file 1 [file Data_Sheet_1.PDF]

Supplementary material 1. Sources reviewed during the evidence and context review and their contribution to resource development

Domain reviewed	Sources	Purpose	Contribution to resource development
Scientific and public health evidence	Scientific literature; French epidemiological and surveillance data, including Santé publique France	Identify current scientific knowledge and public health priorities	Updated scientific content; French epidemiological context; national/local examples
School curricula	Bulletin official Programmes scolaires | Ministère de l'Education nationale; information on forthcoming curriculum developments from the Ministry of National Education	Identify curriculum entry points, learning objectives and expected competencies	Alignment of activities with curriculum requirements and learning objectives
Existing educational resources	Eduscol e-Bug - Des ressources éducatives sur les microbes et les infections | éduscol | Ministère de l'Éducation nationale | Direction générale de l'enseignement scolaire; exploratory web search for One Health educational resources	Identify available resources and potential gaps	Identification of needs not covered by existing resources and avoidance of unnecessary duplication
Teachers' perspectives	FG1; educational resources reported or identified by teachers	Identify perceived gaps, needs and classroom constraints	Resource format, duration, level of complexity, pedagogical approach and implementation requirements
