# Supplementary material for: Developing evidence-based health education resources: a participatory and iterative approach applied to the one health concept
Source: Front Public Health. 2026 Sep 23;14:1912836. doi: 10.3389/fpubh.2026.1912836 (PMC13642583; doi:10.3389/fpubh.2026.1912836)
Supplement: Supplementary file 2 [file Data_Sheet_2.PDF]

Supplementary Material 2. Focus group guide used for prototype optimisation (FG2)

One Health resource development focus group topic guide

Welcome and consent

Information on how this FG will run:

- This group discussion will last 60 minutes
- I don't expect us all to agree on everything we discuss today, but as a ground rule, please be respectful of one another's points of view.
- There are no right or wrong answers with any of the topic matter we will be discussing today – what's important is that you feel you can be as honest as possible.
- I just want to check everyone's expectations of the discussion today. We are here to discuss your feedback on the first draft of the One Health resources. Is this the expectation that everyone has for the focus group today?
- I am planning to record this discussion today. To be clear, this recording is just used to ensure we have an accurate record of what you tell us. It is for internal analytical purposes only and will not be shared with any third-party organisation. Is everyone happy for me to turn on this recording?

Introductions

To kick us off I just want to go around the room and introduce ourselves – please can you tell us your name, role, what grade and what subject you teach. I'll go first, my name is XXX, and XXX…'

1. Have you had time to look through the resource?
2. What are your overall thoughts on the resources that have been created?
3. What do you think about the information sheets?

Content

1. What do you think about the length and content of the introductory information?
   Probe using a & b:
   a. Is there enough content to support you to run the lesson?
   b. Is it straightforward and easy to use/understand?

2. Is the curriculum links satisfactory to you?

3. How well do you think the learning outcomes fit into the Key Stage / Age group?

4. What do you think of the lesson plan?
    a. Do you think that the lesson plan meets the expectations of the learning objectives?
    b. Are the supporting information (illustrations / diagrams/tables) helpful?
    c. How challenging would you find using the lesson plan in your setting? *Probe further if any challenges mentioned*

5. What do you think of the discussion and learning consolidation section of the resource?

6. What do you think about the name / title of the activities?
    a. If there are any you would change, what would you change it to?

7. What are your thoughts on running these activities in the classroom? *Probe using a & b:*
    a. Do you foresee any barriers with the activities? *Probe further if any barriers mentioned and how it can be resolved*
    b. How do you think your students will respond to these activities?

Implementation barriers / facilitators

1. Do you foresee any challenges or barriers, if any, to using this resource in general?

2. Can you see yourself using this resource in your teaching?
    a. Would you recommend this resource to colleagues?

Conclusion

1. Would you require any further information to teach the topic?
   Do you think there is anything missing that would be relevant to add?

2. Is there anything you would remove?

3. Would you need any training or support to use this resource? If so, what kind?
4. Compared with your preferred teaching resources or those you regularly use, what do you think of this resource?

5. Once the final version of the resources on vectors and vector-borne infections / One Health has been developed, would you be willing to test them in your classroom?

Is there anything that we haven't covered that you would like to add about the resource?
